# DiffCoEx: a simple and sensitive method to find differentially coexpressed gene modules

**DOI:** 10.1186/1471-2105-11-497

**Published:** 2010-10-06

**Authors:** Bruno M Tesson, Rainer Breitling, Ritsert C Jansen

**Affiliations:** 1 Groningen Bioinformatics Center, University of Groningen, Kerklaan 30, 9751 NN Haren, the Netherlands; 2 Institute of Molecular, Cell and Systems Biology, College of Medical, Veterinary and Life Sciences, University of Glasgow, Glasgow G12 8QQ, UK; 3 Department of Genetics, University Medical Center Groningen, 9700 RB, Groningen, the Netherlands

## Abstract

**Background:**

Large microarray datasets have enabled gene regulation to be studied through coexpression analysis. While numerous methods have been developed for identifying differentially expressed genes between two conditions, the field of differential coexpression analysis is still relatively new. More specifically, there is so far no sensitive and untargeted method to identify gene modules (also known as gene sets or clusters) that are differentially coexpressed between two conditions. Here, sensitive and untargeted means that the method should be able to construct *de novo *modules by grouping genes based on shared, but subtle, differential correlation patterns.

**Results:**

We present DiffCoEx, a novel method for identifying correlation pattern changes, which builds on the commonly used Weighted Gene Coexpression Network Analysis (WGCNA) framework for coexpression analysis. We demonstrate its usefulness by identifying biologically relevant, differentially coexpressed modules in a rat cancer dataset.

**Conclusions:**

DiffCoEx is a simple and sensitive method to identify gene coexpression differences between multiple conditions.

## Background

There are two major classes of approach to the analysis of gene expression data collected in microarray studies: either one can identify genes that are differentially expressed in different conditions, or the patterns of correlated gene expression (coexpression). Coexpression analysis identifies sets of genes that are expressed in a coordinated fashion, i.e. respond in a similar fashion to the controlled or uncontrolled perturbation present in the experiment. Such coexpression is considered as evidence for possible co-regulation and for membership to common biological processes under the principle of guilt-by-association [1]. When comparing the transcriptome between two conditions, it is a natural step to identify differential coexpression to get an even more informative picture of the dynamic changes in the gene regulatory networks. Changes in the differential coexpression structure of the genes are, for example, a group of genes strongly correlated in one condition but not in the other, or one module correlating to another module in one condition, whereas they are no longer correlated in the other condition. Differential coexpression may indicate rewiring of transcriptional networks in response to disease or adaptation to different environments.

Differential coexpression has been reported in diverse organisms and across various conditions. For example, Fuller et al. [2] reported a differentially coexpressed module in obese mice compared to lean mice; Van Nas et al. [3] found gender-specific coexpression modules; Oldham et al. [4] identified gene modules that were differentially coexpressed between humans and chimpanzees; and Southworth et al. [5] found that aging in mice was associated with a general decrease in coexpression. Differential coexpression patterns associated with diseases have been an important focus of research, see review by De la Fuente et al. [6].

Differential coexpression methods can be divided into two categories that serve distinct purposes: on the one hand, targeted approaches study gene modules that are defined *a priori*, while, on the other hand, untargeted approaches aim at grouping genes into modules on the basis of their differential coexpression status.

A suitable untargeted method for differential coexpression analysis should satisfy the following criteria:

(i) Sensitively detect groups of genes in which the correlation of gene pairs within the group is significantly different between conditions.

(ii) Sensitively detect changes in correlations between two groups of genes even when the within-group correlation is conserved across conditions.

(iii) Allow for simple comparison of more than two conditions.

**Figure 1:**
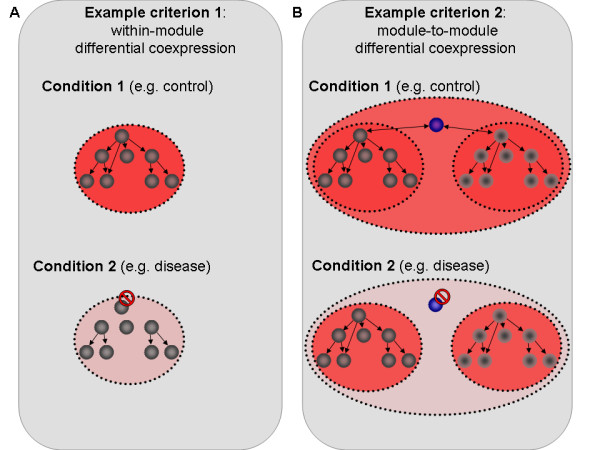
**Illustration of differential coexpression scenarios**. Panel A: A gene network is in a coexpressed state in condition 1 as shown by the red background. In condition 2 an important regulator of that network is now inactive and the module is no longer coexpressed. This scenario is an example of the differential coexpression type described by criterion (i). Panel B: Two pathways are coordinated in condition 1 via an important hub gene (shown in blue) whose inactivity in condition 2 means the two pathways are no longer coexpressed. This exemplifies the module-to-module differential coexpression described by criterion (ii).

Criteria (i) and (ii) are illustrated in Figure 1, which schematically depicts biological scenarios that can give rise to differential coexpression.

Multiple methods have been proposed to identify such large-scale correlation patterns [5,7-12]. However, this early work provided only partial solutions to the problem of differential coexpression since, with one recent exception [5], none of the proposed methods were entirely untargeted. Instead, existing methods can be divided into two categories: targeted and "semi-targeted" approaches. In targeted approaches, pre-defined modules are surveyed for correlation changes between two conditions. For example, Choi et al. [9] proposed a method that focuses on the analysis of modules based on known gene annotations, such as GO categories, and tests the significance of the coexpression changes using a statistical measure known as dispersion. This has the advantage of not requiring the gene sets to be highly correlated in one of the two conditions. However, this method is targeted in that it relies on the study of known functional gene sets and is not able to identify novel, non-annotated modules or modules that would only partially match annotated categories. "Semi-targeted" approaches use classical coexpression methods in one of the conditions to define modules and study whether these modules are also coexpressed in the second condition. DCA (differential clustering analysis) [10] is an example of a method using one of the two conditions as reference, meaning the clusters under consideration are obtained from one condition and then studied in the other condition. In order to avoid bias towards one of the conditions, Ihmels et al. suggested doing a reciprocal analysis, switching the reference and target conditions, while Southworth et al. used a third dataset as reference [5]. A drawback of such "semi-targeted" methods is that the analysis will only focus on groups of genes that emerge as clusters in at least one of the conditions, and will therefore potentially miss more subtle cases. As an example, a weak but significant condition-dependent correlation structure between a group of genes that otherwise belong to distinct, strongly coexpressed and conserved clusters would not be detected by this approach. A first attempt at an untargeted approach was introduced by Southworth et al. [5], who proposed applying hierarchical clustering using the difference in pairwise correlations between both conditions as a similarity metric for two genes. This approach is therefore suited to identifying groups in which the within-group correlation changes (first criterion), but it cannot be applied to the detection of module-to-module correlation differences (second criterion). The field of differential coexpression analysis would therefore benefit from a new, truly untargeted and sensitive method for identifying differentially correlated modules that would satisfy all three criteria.

Here we present a solution to this problem in the form of the DiffCoEx approach for untargeted differential coexpression analysis: a method which applies the powerful tools of Weighted Gene Coexpression Network Analysis (WGCNA) to differential network analysis. We first describe the five steps involved in DiffCoEx and then, to illustrate the method's effectiveness, we present the results of an analysis performed on a publicly available dataset generated by Stemmer et al. [13].

## Algorithm

Our method builds on WGCNA [14,15], which is a framework for coexpression analysis. Identification of coexpression modules with WGCNA follows three steps: first an adjacency matrix is defined between all the genes under consideration based on pair-wise correlations. Then the generalized topological overlap measure [16] is computed from the adjacency matrix and converted into a dissimilarity measure. Finally, using this dissimilarity measure, hierarchical clustering is applied, followed by tree cutting using either a static or a dynamic height cut. The resulting clusters form modules of genes in which all members are strongly inter-correlated.

The principle of DiffCoEx is to apply WGCNA to an adjacency matrix representing the correlation changes between conditions. DiffCoEx clusters genes using a novel dissimilarity measure computed from the topological overlap [16] of the correlation changes between conditions. Intuitively, the method groups two genes together when their correlations to the same sets of genes change between the different conditions. The complete process of our differential coexpression analysis comprises five steps, described below. The notation *X *designates a square matrix with the dimension of the number of genes considered and *x*_*ij *_is used to define the element of *X *at row *i *and column *j*.

### Step 1

Build adjacency matrix *C*^*[k] *^within each condition *k *as the correlation for all pair of genes (*i,j*):

$$C^{\left[k\right]}:c_{ij}^{\left[k\right]}\;=\;cor(gene_{i},gene_{j})$$

In this step, different correlation measures can be used, such as the Pearson or Spearman coefficient.

### Step 2

Compute matrix of adjacency difference:

$$D:d_{ij}\;={\left(\sqrt{\frac{1}{2}\left|sign\;(c_{ij}^{\left[1\right]})\;*\;{\left(c_{ij}^{\left[1\right]}\right)}^{2}\;-sign(c_{ij}^{\left[2\right]})\;*\;{\left(c_{ij}^{\left[2\right]}\right)}^{2}\right|}\right)}^{\beta }$$

In this matrix, high values of *d*_*ij *_indicate that the coexpression status of *gene*_*i *_and *gene*_*j *_changes significantly between the two conditions. The correlation change is quantified as the difference between signed squared correlation coefficients so that changes in correlation which are identical in terms of explained variance (*r*^*2*^) are given the same weight. This adjacency matrix is defined such that it only takes values between 0 and 1. The soft threshold parameter *β *is taken as a positive integer and is used to transform the correlation values so that the weight of large correlation differences is emphasized compared to lower, less meaningful, differences. *β *should be regarded as a tuning parameter, and in practice it is advisable to try different values of *β*. In WGCNA, it is recommended to choose *β *so that the resulting coexpression network follows an approximate scale-free topology [14]. However the "scale-free" topology nature of biological networks has been disputed [17], and another way is to consider the soft threshold parameter as a stringency parameter: using high values of *β *means putting less emphasis on smaller changes in correlation, and therefore being more statistically stringent. Accordingly, since larger sample sizes come with higher statistical significance of small correlation changes, smaller values of the soft threshold can be used as the sample size increases. In practice, we view the soft threshold parameter as a tuning parameter, and we always check the significance of the result afterwards, both statistically and using biological criteria relevant in each specific study.

### Step 3

Derive the Topological Overlap [16] based dissimilarity matrix *T *from the adjacency change matrix *D*.

$$T:t_{ij}=1-\frac{\sum_{k}\left(d_{ik}\;\;d_{kj}\right)+d_{ij}}{\min\left(\sum_{k}d_{ik},\sum_{k}d_{jk}\right)+1-d_{ij}}$$

The use of the topological overlap measure to construct a dissimilarity metrics allows the identification of genes that share the same neighbors in the graph formed by the differential correlation network as defined by the adjacency matrix created in Step 2. Intuitively, a low value of *t*_*ij *_(high similarity) means that *gene*_*i *_and *gene*_*j *_both have significant correlation changes with the same large group of genes. This group of genes constitutes their "topological overlap" in the differential correlation network and may, or may not, include *gene*_*i *_and *gene*_*j*_. This property allows DiffCoEx to satisfy both criteria (i) and (ii) as stated earlier. On the one hand, if *gene*_*i *_and *gene*_*j *_are part of a module of genes coexpressed in only one condition (criterion (i), illustrated in Figure 1A), then the topological overlap between *gene*_*i *_and *gene*_*j *_in the difference network consists of all the genes within that module. On the other hand, if *gene*_*i *_and *gene*_*j *_are equally inter-correlated in both conditions but correlate with the genes in a distinct module in only one condition (criterion (ii), illustrated in Figure 1B), then the topological overlap between *gene*_*i *_and *gene*_*j *_in the difference network consists of the genes in that other module. In both cases *gene*_*i *_and *gene*_*j *_will therefore be grouped together: in the first case forming a differentially correlated module, and in the second case forming a module with differential module-to-module correlation with another group of genes.

We note that since the adjacency matrix takes values between 0 and 1, the dissimilarity matrix computed here also takes values between 0 and 1, as shown in [14].

### Step 4

The dissimilarity matrix *T *is used as input for clustering and modules are identified.

The clustering can be done using standard hierarchical clustering with average linkage, followed by module extraction from the resulting dendrogram, either using a fixed cut height or with more elaborate algorithms such as the dynamicTreeCut [18]. Alternative clustering techniques, such as Partitioning Around Medoids (PAM) [19], may be used in this step.

### Step 5

Assess the statistical significance of coexpression changes.

This is necessary because DiffCoEx uses user-defined parameters: the soft threshold *β *used to transform the adjacency matrix in Step 2 and the clustering parameters in Step 4 (tree cutting settings, for example). Unsuitable settings may lead to the detection of clusters with non-significant differential coexpression.

The statistical significance of differential coexpression can be assessed using a measure of the module-wise correlation changes such as the dispersion statistic [9], the t-statistic [12], or the average absolute correlation. Permutations or simulations of the data can be used to generate a null distribution of those statistics by providing estimates of the extent of differential correlation that can be expected to occur by chance. An example of implementing a permutation procedure to assess the significance of differential coexpression using the dispersion statistics is presented in Additional File 1.

### Variants

#### Extending the DiffCoEx method to multiple conditions

This method can easily be extended to the study of differential coexpression over more than two conditions. The only required change is in Step 2, where the matrix of adjacency differences should be replaced with the following: supposing we have calculated C[1],...,C^[k]^,...,C^[n] ^the correlation matrices for gene pairs in each of the *n *different conditions:

$$D:\;d_{ij}={\left(\sqrt{\frac{1}{n-1}\sum_{k}\frac{\left|sign(c_{ij}^{\left[k\right]})\;*\;{\left(c_{ij}^{\left[k\right]}\right)}^{2}-\;c_{ij}^{\left[0\right]}\right|}{2}}\right)}^{\beta }$$

where

$$c_{ij}^{\left[0\right]}\;=\;\frac{1}{n}\;\sum_{k}\left(sign(c_{ij}^{\left[k\right]})\;*\;{\left(c_{ij}^{\left[k\right]}\right)}^{2}\right)$$

For two conditions, one can verify that this formulation is equivalent to that proposed earlier in Step 2.

#### A less sensitive variant to detect more striking patterns

If one is interested in picking up only coexpression changes that affect genes forming highly coexpressed modules in at least one of the conditions, the formula in Step 2 can be adapted so that the method uses the difference between the two transformed correlation matrices (with the soft threshold parameter *β*) as shown below:

$$D:d_{ij}\;=\frac{1}{2}\left|sign\;(c_{ij}^{\left[1\right]})\;*\;{\left(c_{ij}^{\left[1\right]}\right)}^{\beta }\;-sign\;(c_{ij}^{\left[2\right]})\;*\;{\left(c_{ij}^{\left[2\right]}\right)}^{\beta }\right|$$

This will make the method less sensitive to subtle coexpression changes, but may help in extracting more strikingly differentially coexpressed modules.

#### Variant without the topological overlap

As with WGCNA, the use of a topological overlap-based metrics makes the approach very sensitive, since it considers the correlation changes to all other genes to determine the similarity between two genes. The method can be simplified by replacing the dissimilarity matrix *T *of Step 4 by a dissimilarity measure derived directly from the adjacency matrix *D*:

$$T_{alt}=\text{1}–D$$

This will make DiffCoEx focus only on within-module differential coexpression (criteria (i)) and not on module-to-module differential coexpression (criteria (ii)). This variant is computationally more efficient since the topological overlap computation is omitted.

## Results

We present here the results of our method as used on a previously published dataset. We identify modules of genes that are differentially coexpressed and, by using gene set enrichment analysis, we provide evidence for their biological relevance.

### Dataset

Our dataset (Gene Expression Omnibus GEO GSE5923) contains Affymetrix gene expression profiles of renal cortex outer medulla in wild-type- and Eker rats treated with carcinogens. The dataset is a time course as the rats were treated with Aristolochic Acid (AA) or Ochratoxin A (OTA), respectively, for 1, 3, 7 or 14 days. In total, the dataset consists of 84 arrays measuring 15,923 probe sets. Details about the experimental settings are available in the original paper [13].

Eker rats are predisposed to renal tumor because they are heterozygous for a loss-of-function mutation in the tuberous sclerosis 2 (*Tsc2*) tumor suppressor gene. Stemmer et al. [13] compared the transcriptional responses of the rats to the carcinogens and found that the expression levels of genes belonging to a number of cancer-related pathways were affected differently in the mutant compared to the wild-type rats. In our re-analysis of the data, we switched the focus from differential expression to differential coexpression in an attempt to identify functional modules responding to carcinogen treatment with a different coexpression signature in mutant Eker rats compared to wild type rats.

### Analysis

We applied the DiffCoEx method to the quantile normalized data [20]. A duplicate set of 12 controls present only for Eker rats was discarded in order to have a symmetric experimental setting among wild-type- and Eker rats. We used the Spearman rank correlation in order to reduce sensitivity to outliers, and the hierarchical clustering and module assignment was performed using dynamicTreeCut [18]. The detailed algorithm and R code used in this analysis are given in Additional File 1.

### Findings

**Figure 2:**
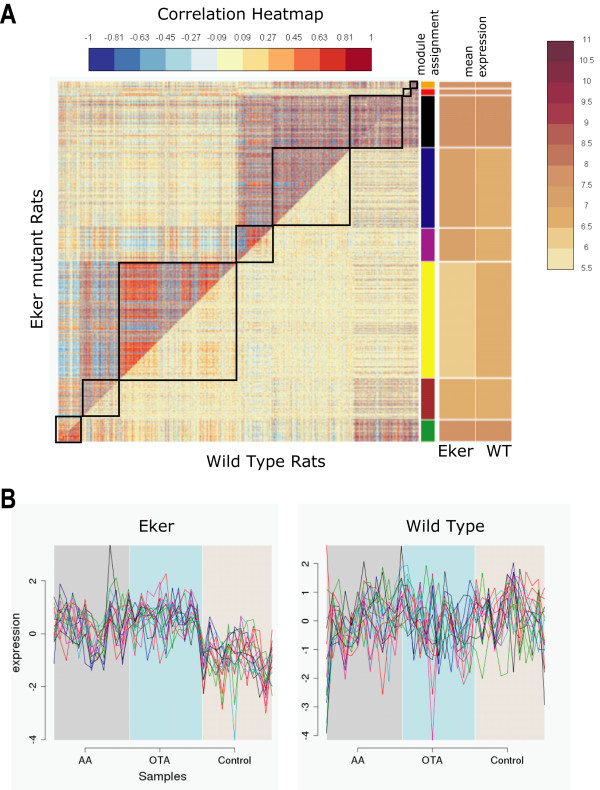
**Differentially coexpressed modules between carcinogen-treated Eker rats and wild-type rats**. Panel A: Comparative correlation heat map. The upper diagonal of the main matrix shows a correlation between pairs of genes among the Eker mutant rats (the red color corresponds to positive correlations, blue to negative correlations). The lower diagonal of the heat map shows a correlation between the same gene pairs in the wild-type controls. Modules are identified in the heat map by black squares and on the right side of the heat map by a color bar. The brown bands on the right side indicate the mean expression of the modules in the Eker rats (first column) and the wild-type rats (second column); darker colors indicate higher mean expression levels. Panel B: Expression variation (scaled) in the Eker mutants (left) and the wild-type rats (right) of the genes in the yellow module which are annotated in KEGG with "pancreatic cancer". In the Eker rats the variation of these genes is tightly correlated, whereas for the wild-type rats it is much more random.

The results of the analysis are summarized in Figure 2A. We identified a total of 8 differentially coexpressed modules comprising a total of close to 1800 genes (1887 probe sets, 1796 unique genes). The modules were given color names as indicated in Figure 2A. Four of these modules (totaling 1361 genes) were significantly more highly correlated in the mutant Eker rats than in the wild-type rats, while only the red module (36 genes) and, to a lesser extent, the green module (116 genes) follow the opposite pattern. This striking asymmetry might reflect the greater fragility of the Eker rats to carcinogens: in Eker rats, treatment with carcinogens leads to much more coordinated perturbation of the transcriptome than in wild-type rats.

The cases of the black, orange and green modules illustrate an interesting characteristic of DiffCoEx: the method is able to identify module-to-module correlation changes. Interestingly, the black module is not differentially correlated in the wild-type rats compared to the Eker rats. Instead, what qualifies the black module as a differentially coexpressed module is its very significant drop in correlation with the genes in the blue and purple modules in the wild-type rats compared to the Eker mutants (see Figure 2A). Similar patterns can be observed for the orange and green modules. This property makes DiffCoEx a sensitive approach for detecting any type of large-scale correlation change.

Following Choi et al. [9], significance of the coexpression differences was assessed by comparing the dispersion index values of each module in the data with the null distribution obtained from permuted (scaled) data (see Additional File 1 for details and Additional File 2: **Figure S1 **for an overview of the permutation results). In 1000 permutations, none of the blue, brown, purple, red or yellow modules obtained as high a dispersion value as that obtained from the non-permuted data, indicating a significance *p*-value < 0.001. Module-to-module coexpression changes were tested by assessing the significance of the correlation changes between the genes from each possible module pair, using a similar "module-to-module" dispersion measure and generating null distributions from the same permutation approach. Additional File 2: **Figure S1 **shows that the coexpression change between the black and blue modules, for example, is highly significant since no permutation yielded as high a dispersion value.

In the next step, the biological significance of the modules was surveyed using gene-set enrichment analysis. We submitted each of the modules to GeneTrail [21] and identified many significantly over-represented GO or KEGG terms among the gene annotations. A subset of some of the most interesting findings is presented in Table 1, while complete lists are available as Additional File 3. Interestingly, the black module was enriched for genes involved in "response to xenobiotics", while the blue module contained many genes associated with "metabolic processes". Finally, the yellow module was strongly enriched for genes known to be involved in cancer pathogenesis.

**Table 1:** Annotations enriched in differentially coexpressed modules

Module	Category	Subcategory	Expected		Observed	fdr
**Black**	KEGG	Metabolism of xenobiotics by cytochrome P450	1.367		12	<0.001
	KEGG	Metabolic pathways	22.494		40	<0.001
	GO	Glutathione transferase activity	0.364		9	<0.001
**Blue**	KEGG	Lysosome	3.373		12	0.008
	KEGG	Metabolic pathways	31.541		48	0.026
	GO	Mitochondrion	35.764		67	<0.001
**Brown**	GO	Intracellular transport	8.481		22	0.038
**Green**	GO	Mitochondrion	10.234		26	0.003
	GO	Oxidation reduction	4.015		15	0.003
**Orange**	GO	Xenobiotic metabolic process	0.079		5	<0.001
**Purple**	No significant enrichment					
**Red**	KEGG	Endometrial cancer	0.201		3	0.015
**Yellow**	KEGG	Pancreatic cancer	3.344		14	<0.001
	KEGG	Renal cell carcinoma	3.702		10	0.043
	KEGG	Pathways in cancer	14.75		27	0.022
	GO	Protein localization	33.676		64	<0.001
	GO	Melanosome	2.995		11	0.009
	GO	Cell projection	33.886		59	0.002
	GO	Small GTPase mediated signal transduction	14.342		31	0.003

Selected annotations enriched among the genes of each differentially coexpressed modules and associated false discovery rates (fdr). The over-representation analysis was conducted using GeneTrail. The complete results are available in Additional File 3.

In Figure 2B, the expression data for the 13 genes of the yellow module, which were associated with the "pancreatic cancer" KEGG annotation, illustrate what differential coexpression is: a difference in the coordination of the variation of a group of genes between two conditions. In the Eker rats, these cancer genes show coordinated variation, whereas in the wild-type rats this coordination is absent.

### Implementation

This analysis was carried out using the R statistical package with the WGCNA [15] library, on a Linux computer with 128 GB physical memory. Large memory (around 10 GB) is required to compute correlation matrices for over 10,000 genes. For module definition, hierarchical clustering was combined with dynamicTreeCut [18] using a minimum size of 20 genes. Details of the process and code can be found in Additional File 1.

## Discussion and conclusions

The method we present here has the advantage of comparing two (or more) datasets in a global, unbiased and unsupervised manner. It represents a major improvement over earlier two-way comparisons, in which clustering was first performed in one condition and the coexpression of the genes in the resulting clusters was then assessed in the other condition. Moreover, DiffCoEx is very sensitive because (i) it does not require differentially coexpressed modules to be detected as coherent, coexpressed modules in one of the two conditions; instead, only the difference in coexpression is considered to define the module; and (ii) it can identify all types of large-scale correlation changes, including module-to-module correlation changes. Using a simulation study (see Additional File 4), we demonstrate examples of differential coexpression patterns that can be uncovered using DiffCoEx but that were missed by existing approaches.

Differential coexpression provides information that would be missed using classical methods focusing on the identification of differentially expressed genes. For example, as Figure 2A shows, many of the differentially coexpressed clusters display few differences between the two conditions in terms of mean overall expression. This indicates that the changes in correlation that we observed cannot be explained by the genes being not expressed, and therefore not correlated in one of the two conditions.

Differential coexpression may be caused by different biological mechanisms. For example, a group of genes may be under the control of a common regulator (e.g. a transcription factor or epigenetic modification) that is active in one condition, but absent in the other condition. In such a case, the correlation structure induced by variation in the common regulator would only be present in the first condition. Another possible interpretation relates to the presence or absence of variation in some factors driving a gene module. To observe correlation of a group of genes responding to a common factor, this factor needs to vary. In the absence of variation of the driving factor, no correlation can be observed, even though the actual biological links that form the network are not altered. It is therefore important to ensure that the perturbations which give rise to variation within each condition are: (i) biologically relevant (as opposed to batch effects, for example) and (ii) comparable in nature and amplitude.

DiffCoEx provides a simple and efficient approach to study how different sample groups respond to the same perturbations. These perturbations can be either well characterized and controlled, or stochastic and unknown. In our example analysis, on top of random physiological fluctuations present in any dataset, there was a controlled perturbation induced by the time-course treatment with different carcinogens present. Since the carcinogen treatment is a controlled experimental factor, it is possible to use classical methods to study the transcriptomic changes it induces rather than using DiffCoEx. However, a fundamental advantage of using DiffCoEx in such a case is that it requires no model assumptions and is a quick and efficient approach. Differential coexpression approaches are even more useful when the variation among the samples in one condition is caused by uncontrolled factors, whose effects cannot easily be dissected. A typical example would be genetic variation present in a natural population or an experimental cross. DiffCoEx constitutes a valuable tool of broad applicability now that such genetic studies are becoming increasingly important for studying gene regulatory networks [22-24].

## Authors' contributions

BMT designed and implemented the algorithm, analyzed the results and drafted the manuscript. RB and RCJ directed the project and revised the manuscript. All authors read and approved the final manuscript.

## Supplementary Material

Additional file 1 **Step-by-step R analysis for applying DiffCoEx**. This file contains the documented R source code used to perform the analysis described in the main text as well as the simulation study described in Additional file 4. Click here for file

Additional file 2 **Significance assessment of module-to-module coexpression changes using permutations**. This figure summarizes the results of the significance analysis. 1000 permutations of the samples between the two conditions were performed, and for each of the permuted datasets, the dispersion value (a measure of correlation change for groups of genes) was computed for each module, and for every possible module pair. The number of permutations yielding a higher dispersion value than that of the original data was recorded and is displayed in this figure. The figure, for example, indicates that the within-module dispersion value for the black module reached a higher value with permuted data than with original data 249 times. The within-module coexpression change was therefore not significant (*p *= 0.249) for the black module and this is indicated with a light grey shading. Similarly, the figure shows that no permutations reached as high a value as the original data for the purple to black dispersion, meaning that the black module was significantly differentially coexpressed with the purple module, and this is indicated with dark grey shading. Click here for file

Additional file 3 **Differentially coexpressed modules and enrichment analysis results**. This Excel file has separate sheets for the gene lists for each of the differentially coexpressed modules and the results of the enrichment analysis conducted using GeneTrail. Click here for file

Additional file 4 **Simulation study showing the sensitivity of DiffCoEx**. This file details the result of a simulation study performed to illustrate a scenario in which DiffCoEx will outperform other, less sensitive, methods. Click here for file
