# Supplementary material for: DiffCoEx: a simple and sensitive method to find differentially coexpressed gene modules
Source: BMC Bioinformatics. 2010 Oct 6;11:497. doi: 10.1186/1471-2105-11-497 (PMC2976757; doi:10.1186/1471-2105-11-497)
Supplement: Additional file 1 — Step-by-step R analysis for applying DiffCoEx. This file contains the documented R source code used to perform the analysis described in the main text as well as the simulation study described in Additional file 4. [file 1471-2105-11-497-S1.DOC]

**Supplementary Information: R script used for analysis**

Note: This supplementary file is organized in four sections:

1. Data Loading and preprocessing
2. DiffCoEx analysis
3. Significance testing through permutations
4. Simulation study
5. Supporting functions

The fifth section contains the source code for the functions used in the second and third sections (*e.g.* for plotting), the code contained there should therefore be loaded in R prior to running the DiffCoEx analysis of step 2.

**1. Data Loading and Preprocessing**

#required libraries

#WGCNA package can be found at

#http://www.genetics.ucla.edu/labs/horvath/CoexpressionNetwork/Rpackages/WGCNA

library(WGCNA) ###used for topological overlap calculation and clustering steps

library(RColorBrewer) ###used to create nicer colour palettes

library(preprocessCore) ###used by the quantile normalization function

#Note: the data can be downloaded from the Gene Expression Omnibus

# http://www.ncbi.nlm.nih.gov/sites/GDSbrowser?acc=GDS2901

data<-as.matrix(read.csv(file="GDS2901.soft",skip=166,row.names=1,sep="\t",header=T))

data<-data[-15924,]

rawData<-matrix(as.numeric(data[,-1]),nrow=15923)

dimnames(rawData)<-dimnames(data[,-1])

#we create an annotation matrix containing the matches between probesets and gene names

anno<-as.matrix(data[-2475,1])

normData<-normalize.quantiles(log2(rawData))

dimnames(normData)<-dimnames(rawData)

#we remove the probeset at index 2475 because

#after quantile normalization it has zero variance

#(the probeset has the highest signal of all samples)

normData<-normData[-2475,]

datC1<-t(normData[,c(1:12,25:36,37:48)]) ### these samples correspond to the Eker mutants.

# Note that since the Eker mutants have two sets of 12 control samples (13:24 and 37:48)

# we discard one to have a symmetric perturbation (carcinogenic vs control) between the two conditions (Eker mutants vs wild-types)

datC2<-t(normData[,49:84]) ###those samples correspond to the wild-types

**2. Applying DiffCoEx**

beta1=6 #user defined parameter for soft thresholding

AdjMatC1<-sign(cor(datC1,method="spearman"))*(cor(datC1,method="spearman"))^2

AdjMatC2<-sign(cor(datC2,method="spearman"))*(cor(datC2,method="spearman"))^2

diag(AdjMatC1)<-0

diag(AdjMatC2)<-0

collectGarbage()

dissTOMC1C2=TOMdist((abs(AdjMatC1-AdjMatC2)/2)^(beta1/2))

collectGarbage()

#Hierarchical clustering is performed using the Topological Overlap of the adjacency difference as input distance matrix

geneTreeC1C2 = flashClust(as.dist(dissTOMC1C2), method = "average");

# Plot the resulting clustering tree (dendrogram)

png(file="hierarchicalTree.png",height=1000,width=1000)

plot(geneTreeC1C2, xlab="", sub="", main = "Gene clustering on TOM-based dissimilarity",labels = FALSE, hang = 0.04);

dev.off()

#We now extract modules from the hierarchical tree. This is done using cutreeDynamic. Please refer to WGCNA package documentation for details

dynamicModsHybridC1C2 = cutreeDynamic(dendro = geneTreeC1C2, distM = dissTOMC1C2,method="hybrid",cutHeight=.996,deepSplit = T, pamRespectsDendro = FALSE,minClusterSize = 20);

#Every module is assigned a color. Note that GREY is reserved for genes which do not belong to any differentially coexpressed module

dynamicColorsHybridC1C2 = labels2colors(dynamicModsHybridC1C2)

#the next step merges clusters which are close (see WGCNA package documentation)

mergedColorC1C2<-mergeCloseModules(rbind(datC1,datC2),dynamicColorsHybridC1C2,cutHeight=.2)$color

colorh1C1C2<-mergedColorC1C2

#reassign better colors

colorh1C1C2[which(colorh1C1C2 =="midnightblue")]<-"red"

colorh1C1C2[which(colorh1C1C2 =="lightgreen")]<-"yellow"

colorh1C1C2[which(colorh1C1C2 =="cyan")]<-"orange"

colorh1C1C2[which(colorh1C1C2 =="lightcyan")]<-"green"

# Plot the dendrogram and colors underneath

png(file="module_assignment.png",width=1000,height=1000)

plotDendroAndColors(geneTreeC1C2, colorh1C1C2, "Hybrid Tree Cut",dendroLabels = FALSE, hang = 0.03,addGuide = TRUE, guideHang = 0.05,main = "Gene dendrogram and module colors cells")

dev.off()

#We write each module to an individual file containing affymetrix probeset IDs

modulesC1C2Merged<-extractModules(colorh1C1C2,datC1,anno,dir="modules",file_prefix=paste("Output","Specific_module",sep=''),write=T)

write.table(colorh1C1C2,file="module_assignment.txt",row.names=F,col.names=F,quote=F)

#We plot to a file the comparative heatmap showing correlation changes in the modules

#The code for the function plotC1C2Heatmap and others can be found below under the Supporting Functions section

plotC1C2Heatmap(colorh1C1C2,AdjMatC1,AdjMatC2, datC1, datC2)

png(file="exprChange.png",height=500,width=500)

plotExprChange(datC1,datC2,colorh1C1C2)

dev.off()

**3. Permutation procedure for significance testing**

#This function computes the dispersion value that

#quantifies the change in correlation between two conditions

#for pair of genes drawn from module c1 and module c2

# in case c1 = c2, the function quantifies the differential coexpression in c1.

#cf Choi and Kendziorski 2009

dispersionModule2Module<-function(c1,c2,datC1,datC2,colorh1C1C2)

{

if (c1==c2)

{

difCor<-(cor(datC1[,which(colorh1C1C2 == c1)],method="spearman")-

cor(datC2[,which(colorh1C1C2 == c1)],method="spearman"))^2

n<-length(which(colorh1C1C2 ==c1))

(1/((n^2 -n)/2)*(sum(difCor)/2))^(.5)

}

else if (c1!=c2)

{

difCor<-(cor(datC1[,which(colorh1C1C2 == c1)],datC1[,which(colorh1C1C2==c2)],method="spearman")-

cor(datC2[,which(colorh1C1C2 == c1)],datC2[,which(colorh1C1C2==c2)],method="spearman"))^2

n1<-length(which(colorh1C1C2 ==c1))

n2<-length(which(colorh1C1C2 ==c2))

(1/((n1*n2))*(sum(difCor)))^(.5)

}

}

# we generate a set of 1000 permuted indexes

permutations<-NULL

for (i in 1:1000)

{

permutations<-rbind(permutations,sample(1:(nrow(datC1)+nrow(datC2)),nrow(datC1)))

}

# we scale the data in both conditions to mean 0 and variance 1.

d<-rbind(scale(datC1),scale(datC2))

# This function calculates the dispersion value of a module to module coexpression change on permuted data

permutationProcedureModule2Module<-function(permutation,d,c1,c2,colorh1C1C2)

{

d1<-d[permutation,]

d2<-d[-permutation,]

dispersionModule2Module(c1,c2,d1,d2,colorh1C1C2)

}

#We compute all pairwise module to module dispersion values, and generate a null distribution from permuted scaled data

dispersionMatrix<-matrix(nrow=length(unique(colorh1C1C2))-1,ncol=length(unique(colorh1C1C2))-1)

nullDistrib<-list()

i<-j<-0

for (c1 in setdiff(unique(colorh1C1C2),"grey"))

{

i<-i+1

j<-0

nullDistrib[[c1]]<-list()

for (c2 in setdiff(unique(colorh1C1C2),"grey"))

{

j<-j+1

dispersionMatrix[i,j]<-dispersionModule2Module(c1,c2,datC1,datC2,colorh1C1C2)

nullDistrib[[c1]][[c2]]<-apply(permutations,1,permutationProcedureModule2Module,d,c2,c1,colorh1C1C2)

}

}

#We create a summary matrix indicating for each module to module

#differential coexpression the number of permuted data yielding

#an equal or higher dispersion.

permutationSummary<-matrix(nrow=8,ncol=8)

colnames(permutationSummary)<-setdiff(unique(colorh1C1C2),"grey")

rownames(permutationSummary)<-setdiff(unique(colorh1C1C2),"grey")

for (i in 1:8) { for (j in 1:8) {permutationSummary[i,j]<-length(which(nullDistrib[[i]][[j]] >= dispersionMatrix[i,j]))}}

#We plot the result (cf supplementary figure 1)

plotMatrix(permutationSummary)

**4. Simulation study**

#Loading required libraries.

library(RColorBrewer)

library(WGCNA)

library(coXpress)

#number of samples in each condition

n_samples<-100

#number of non clustered genes

n_others<-800

#initializing the pseudo-random generator in order to be able to replicate the simulation result

set.seed(12345)

#The following function simulates expression data for one module in two conditions.

#The function creates a sub-modular structure within the module by randomly adding correlation into subgroups of size 'size_parts' in a number of runs defined by 'partitionning'

#The correlation is introduced by adding factors whose variance can be defined with parameters:

# v_C1 and v_C2 specify the variance explained by the module main homogenous correlation

# v_within_C1 and v_within_C2 specify variance explained by the submodular correlation

# v_rdm_C1 and v_rdm_C2 specify additional random variance

#The output is a list with two matrix components corresponding to the expression of the modules in both conditions

generateModule<-function( n_genes=100, n_samples=n_samples, size_parts=25, partitionning=3, v_C1=3,v_C2=3, v_within_C1=3, v_within_C2=3, v_rdm_C1=1, v_rdm_C2=1)

{

module_main_c1<-rnorm(n_samples,0,1)

module_main_c2<-rnorm(n_samples,0,1)

module_random_c1<-matrix(rnorm(n_samples*n_genes,0,1),nrow=n_samples)

module_random_c2<-matrix(rnorm(n_samples*n_genes,0,1),nrow=n_samples)

module_sub_c1<-matrix(rep(0,n_samples*n_genes),ncol=n_genes)

module_sub_c2<-matrix(rep(0,n_samples*n_genes),ncol=n_genes)

for ( i in 1:partitionning)

{

n_parts<-n_genes/size_parts

parts<-NULL

for( j in 1:n_parts)

{

parts<-rbind( parts, sample(setdiff(1:n_genes,as.numeric(parts)), size=size_parts, replace=F))

module_sub_c1[,parts[j,]]<-module_sub_c1[,parts[j,]]+rnorm(n_samples,0,1)

module_sub_c2[,parts[j,]]<-module_sub_c2[,parts[j,]]+rnorm(n_samples,0,1)

}

}

module_expr_c1<-scale( v_within_C1^.5 * scale(module_sub_c1) + v_C1^.5 * as.vector(scale(module_main_c1)) +
v_rdm_C1^.5 * scale(module_random_c1))

module_expr_c2<-scale(v_within_C2^.5 * scale(module_sub_c2) + v_C2^.5 * as.vector(scale(module_main_c2))
+ v_rdm_C2^.5 * scale(module_random_c2))

result<-list()

result$c1<-module_expr_c1

result$c2<-module_expr_c2

result

}

#Simulating the expression data: modules 1 to 4 are stable.

tmp<-generateModule(n_genes=100,n_samples=n_samples,size_parts=25,partitionning=2,v_C1=.4,v_C2=.5,v_within_C1=.4,v_within_C2=.5,v_rdm_C1=.1,v_rdm_C2=.1)

module1C1<-tmp$c1

module1C2<-tmp$c2

tmp<-generateModule(n_genes=100,n_samples=n_samples,size_parts=25,partitionning=2,v_C1=.4,v_C2=.5,v_within_C1=.4,v_within_C2=.5,v_rdm_C1=.1,v_rdm_C2=.1)

module2C1<-tmp$c1

module2C2<-tmp$c2

tmp<-generateModule(n_genes=100,n_samples=n_samples,size_parts=25,partitionning=2,v_C1=.4,v_C2=.5,v_within_C1=.4,v_within_C2=.5,v_rdm_C1=.1,v_rdm_C2=.1)

module3C1<-tmp$c1

module3C2<-tmp$c2

tmp<-generateModule(n_genes=100,n_samples=n_samples,size_parts=25,partitionning=2,v_C1=.4,v_C2=.5,v_within_C1=.4,v_within_C2=.5,v_rdm_C1=.1,v_rdm_C2=.1)

module4C1<-tmp$c1

module4C2<-tmp$c2

#Module 6 is differentially coexpressed: v_C1 is chosen as .5 and v_C2 as 0

tmp<-generateModule(n_genes=100,n_samples=n_samples,size_parts=25,partitionning=2,v_C1=.5,v_C2=0,v_within_C1=.3,v_within_C2=.3,v_rdm_C1=.2,v_rdm_C2=.7)

module6C1<-tmp$c1

module6C2<-tmp$c2

#Module 7 and module 8 have module-to-module differential coexpression but no within-module differential coexpression

# the parameters here are chosen to compensate the correlation introduced later on for condition 2 within the modules

# the module-to-module differential coexpression is introduced later with 'seed2'

tmp<-generateModule(n_genes=100,n_samples=n_samples,size_parts=25,partitionning=2,v_C1=.67,v_C2=0,v_within_C1=.3,v_within_C2=.3,v_rdm_C1=.03,v_rdm_C2=.7)

module7C1<-tmp$c1

module7C2<-tmp$c2

tmp<-generateModule(n_genes=100,n_samples=n_samples,size_parts=25,partitionning=2,v_C1=.67,v_C2=0,v_within_C1=.3,v_within_C2=.3,v_rdm_C1=.03,v_rdm_C2=.7)

module8C1<-tmp$c1

module8C2<-tmp$c2

#The other genes do not belong to any modules and are simulated as random

otherGenesC1<-matrix(rnorm(n_others*n_samples,0,1),nrow=n_samples)

otherGenesC2<-matrix(rnorm(n_others*n_samples,0,1),nrow=n_samples)

#Module 5 is constitutes by genes that are parts of Modules 1,2,3 and 4

# and is differentially coexpressed: we add a seed vector explaining 40% of the variance in condition 1

# and a random noise expression with equal variance in condition 2.

seed1<-as.vector(.82*scale(rnorm(n_samples,0,1)))

module1C1[,1:25]<-module1C1[,1:25]+seed1

module2C1[,1:25]<-module2C1[,1:25]+seed1

module3C1[,1:25]<-module3C1[,1:25]+seed1

module4C1[,1:25]<-module4C1[,1:25]+seed1

module1C2[,1:25]<-module1C2[,1:25]+.82*scale(matrix(rnorm(n_samples*25,0,1),nrow=n_samples))

module2C2[,1:25]<-module2C2[,1:25]+.82*scale(matrix(rnorm(n_samples*25,0,1),nrow=n_samples))

module3C2[,1:25]<-module3C2[,1:25]+.82*scale(matrix(rnorm(n_samples*25,0,1),nrow=n_samples))

module4C2[,1:25]<-module4C2[,1:25]+.82*scale(matrix(rnorm(n_samples*25,0,1),nrow=n_samples))

# We add the module7-to-module8 coexpression in condition 2 by adding a seed vector explaining 40%

# the total variance in the module and we add a random matrix of equal variance in condition 1

seed2<-as.vector(.82*scale(rnorm(n_samples,0,1)))

module7C1<-module7C1+.82*scale(matrix(rnorm(n_samples*100,0,1),nrow=n_samples))

module8C1<-module8C1+.82*scale(matrix(rnorm(n_samples*100,0,1),nrow=n_samples))

module7C2<-module7C2+seed2

module8C2<-module8C2+seed2

#assembling the total expression matrices

e1<-scale(cbind(module1C1,module2C1,module3C1,module4C1,module6C1,module7C1,module8C1,otherGenesC1))

e2<-scale(cbind(module1C2,module2C2,module3C2,module4C2,module6C2,module7C2,module8C2,otherGenesC2))

#module (color) assignments for each gene as they were simulated

realColors<-c(rep("yellow",25),rep("red",75),rep("yellow",25),rep("black",75),rep("yellow",25),rep("blue",75),rep("yellow",25),rep("green",75),rep("white",100),rep("brown",100),rep("pink",100),rep("grey",n_others))

#randomizing the order of genes

gene_perm<-sample(1:ncol(e1),ncol(e1),replace=F)

e1<-e1[,gene_perm]

e2<-e2[,gene_perm]

realColors<-realColors[gene_perm]

#Running a semi-targeted approach (coXpress)

#clustering in condition 1 (normally we should cluster in both conditions successively,

# but in this simulation it does not add information)

treeC1<-flashClust(as.dist(1-abs(cor(e1,method="spearman"))),method="average")

#assigning modules

coXpress_treecut<-cutree(treeC1,h=.65)

modules_coXpress<-coXpress_treecut

modules_coXpress[which(modules_coXpress %in% names(table(modules_coXpress)[which(table(modules_coXpress)<=20)]))]<-0

coXpress_colors<-labels2colors(modules_coXpress)

#plotting the results

x11();plotDendroAndColors(treeC1, realColors, "Hybrid Tree Cut",dendroLabels = FALSE, hang = 0.03,addGuide = TRUE, guideHang = 0.05,main = "Gene dendrogram and module assignment")

x11();plotDendroAndColors(treeC1,coXpress_colors , "Hybrid Tree Cut",dendroLabels = FALSE, hang = 0.03,addGuide = TRUE, guideHang = 0.05,main = "Gene dendrogram and module assignment")

# Assessing differential coexpression in each modules.

# One can see that only one module (module6) has a large mean difference in correlation

cox <- coXpress(t(rbind(e1,e2)), coXpress_treecut, 1:n_samples,(n_samples+1):(2*n_samples),whichgroups=as.numeric(names(table(coXpress_treecut)[which(table(coXpress_treecut)>=20)])))

cox

#Running DiffCoEx

beta1=1

#calculating signed squared correlation matrices

AdjMatC1<-sign(cor(e1,method="spearman"))*(cor(e1,method="spearman"))^2

AdjMatC2<-sign(cor(e2,method="spearman"))*(cor(e2,method="spearman"))^2

diag(AdjMatC1)<-0

diag(AdjMatC2)<-0

collectGarbage()

#calculating the topological overlap based dissimilarity matrix

dissTOMC1C2=TOMdist((abs(AdjMatC1-AdjMatC2)/2)^(beta1/2))

collectGarbage()

#hierarchical clustering

geneTreeC1C2 = flashClust(as.dist(dissTOMC1C2), method = "average");

#module assignment, in this simple simulation we use a fixed height cut

modules_DiffCoEx<-cutree(geneTreeC1C2,h=.875)

modules_DiffCoEx[which(modules_DiffCoEx %in% names(table(modules_DiffCoEx)[which(table(modules_DiffCoEx)<=20)]))]<-0

DiffCoEx_colors<-labels2colors(modules_DiffCoEx)

#plotting tge results

x11();plotDendroAndColors(geneTreeC1C2, realColors, "Hybrid Tree Cut",dendroLabels = FALSE, hang = 0.03,addGuide = TRUE, guideHang = 0.05,main = "Gene dendrogram and module assignment")

plotC1C2Heatmap(DiffCoEx_colors,AdjMatC1,AdjMatC2, e1, e2,file="result_simulation_DiffCoEx.png")

**5. Supporting functions**

This section contains supporting functions used in the previous section. The code found here should therefore be run into R, prior to performing the analyses described in the previous sections.

##extractModules: a function which uses the module assignment list as input and writes individual files with the probeset ids for each module

extractModules<-function(colorh1,datExpr,anno,write=F,file_prefix="",dir=NULL)

{

module<-list()

if (!is.null(dir))

{

dir.create(dir)

file_prefix=paste(dir,"/",file_prefix,sep="")

}

i<-1

for (c in unique(colorh1))

{

module[[i]]<-(anno[colnames(datExpr)[which(colorh1==c)],1])

if (write) {write.table(rownames(anno)[which(colorh1==c)],file=paste(file_prefix,"_",c,".txt",sep=""),quote=F,row.names=F,col.names=F)}

i<-i+1

}

names(module)<-unique(colorh1)

module

}

##EigenGenes : this is used by the plotting function to display close together similar modules based on their eigen values

getEigenGeneValues<-function(datRef,colorh1,datAll)

{

eigenGenesCoef<-list()

i<-0

for (c in unique(colorh1))

{

i<-i+1

eigenGenesCoef[[i]]<-prcomp(scale(datRef[,which(colorh1 == c)]))$rotation[,1]

}

names(eigenGenesCoef)<-unique(colorh1)

values<-NULL

for( c in unique(colorh1))

{

v<-rbind(datAll)[,which(colorh1 == c)] %*% eigenGenesCoef[[c]]

values<-cbind(values,sign(mean(v))*v)

}

colnames(values)<-unique(colorh1)

values

}

####plotting function for comparative heatmap

plotC1C2Heatmap<-function(colorh1C1C2,AdjMat1C1,AdjMat1C2, datC1, datC2,ordering=NULL,file="DifferentialPlot.png")

{

if (is.null(ordering))

{

h<-hclust(as.dist(1-abs(cor(getEigenGeneValues(datC1[,which(colorh1C1C2!="grey")],colorh1C1C2[which(colorh1C1C2!="grey")],rbind(datC1,datC2)[,which(colorh1C1C2!="grey")])))))

for (c in h$label[h$order])

{

ordering<-c(ordering,which(colorh1C1C2 ==c))

}

}

mat_tmp<-(AdjMat1C1[ordering,ordering])

mat_tmp[which(row(mat_tmp)>col(mat_tmp))]<-(AdjMat1C2[ordering,ordering][which(row(mat_tmp)>col(mat_tmp))])

diag(mat_tmp)<-0

mat_tmp<-sign(mat_tmp)*abs(mat_tmp)^(1/2)

png(file=file,height=1000,width=1000)

image(mat_tmp,col=rev(brewer.pal(11,"RdYlBu")),axes=F,asp=1,breaks=seq(-1,1,length.out=12))

dev.off()

unique(colorh1C1C2[ordering])

}

##This function plots side by side the color bar of module assignments, and the change in mean expression of the modules between the two conditions.

plotExprChange<-function(datC1,datC2, colorhC1C2,ordering=NULL)

{

if (is.null(ordering))

{

h<-hclust(as.dist(1-abs(cor(getEigenGeneValues(datC1[,which(colorh1C1C2!="grey")],colorh1C1C2[which(colorh1C1C2!="grey")],rbind(datC1,datC2)[,which(colorh1C1C2!="grey")])))))

for (c in h$label[h$order])

{

ordering<-c(ordering,which(colorh1C1C2 ==c))

}

}

mycolors<-colorh1C1C2[ordering]

plot(x=0:length(which(mycolors!="grey")),y=rep(1,length(which(mycolors!="grey"))+1),col="white",axes=F,xlab="",ylab="",ylim=c(0,1))

rr=c(244,239,225,215,209,193,181,166,151,130,110)

gg=c(228,204,174,160,146,117,94,58,44,45,45)

bb=c(176,140,109,105,102,91,84,74,70,68,66)

MyColours<-NULL

for ( i in 1:11)

{

MyColours=c(MyColours,rgb(rr[i],gg[i],bb[i],maxColorValue=255) )

}

exprDiff<-NULL

l<-0

for (c in setdiff(unique(mycolors),"grey"))

{

meanC1<-mean(t(datC1)[colnames(datC1)[which(colorh1C1C2 == c)],])

meanC2<-mean(t(datC2)[colnames(datC2)[which(colorh1C1C2 == c)],])

exprDiff<-rbind(exprDiff,c(meanC1,meanC2))

r<-l+length(which(mycolors==c))

rect(l,0.85,r,1,col=c,border=F)

rect(l,0,r,.4,col=MyColours[floor(meanC2*2)-10],border="white",lwd=2)

rect(l,0.4,r,.8,col=MyColours[floor(meanC1*2)-10],border="white",lwd=2)

l<-r

}

exprDiff

}

#plotMatrix is a function used to make **Additional File 2: Figure S1** plot displaying the
# permutation results.

plotMatrix<-function(mat)

{

mat[which(row(mat)>col(mat))]<-1001 image(mat,col=c(gray.colors(4),"white"),breaks=c(0,0.1,50,100,1000,1001),xaxt='n',yaxt='n',xlim=c(-0.2,1.2),ylim=c(-0.2,1.2),bty='n',asp=1)

text(0:(nrow(mat)-1)/(nrow(mat)-1),1.1,rownames(mat),cex=1,col=rownames(mat))

text(-0.15,0:(ncol(mat)-1)/(ncol(mat)-1),colnames(mat),cex=1,col=colnames(mat))

text(apply(matrix(0:(nrow(mat)-1)/(nrow(mat)-1)),1,rep,ncol(mat)),rep(0:(ncol(mat)-1)/(ncol(mat)-1),nrow(mat)),as.numeric(t(mat)),col="white",cex=1.5)

}
