# Supplementary figures and images for: DiffCoEx: a simple and sensitive method to find differentially coexpressed gene modules
Source: BMC Bioinformatics. 2010 Oct 6;11:497. doi: 10.1186/1471-2105-11-497 (PMC2976757; doi:10.1186/1471-2105-11-497)

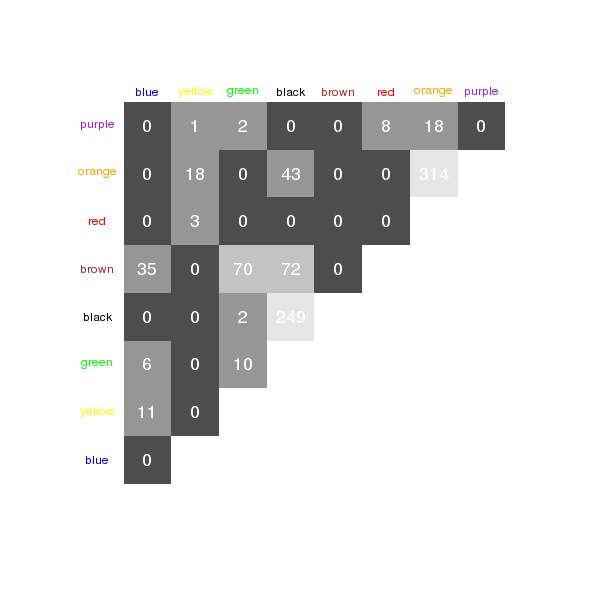

Supplement: Additional file 2 — Significance assessment of module-to-module coexpression changes using permutations. This figure summarizes the results of the significance analysis. 1000 permutations of the samples between the two conditions were performed, and for each of the permuted datasets, the dispersion value (a measure of correlation change for groups of genes) was computed for each module, and for every possible module pair. The number of permutations yielding a higher dispersion value than that of the original data was recorded and is displayed in this figure. The figure, for example, indicates that the within-module dispersion value for the black module reached a higher value with permuted data than with original data 249 times. The within-module coexpression change was therefore not significant (p = 0.249) for the black module and this is indicated with a light grey shading. Similarly, the figure shows that no permutations reached as high a value as the original data for the purple to black dispersion, meaning that the black module was significantly differentially coexpressed with the purple module, and this is indicated with dark grey shading. [file 1471-2105-11-497-S2.PNG]
