# Supplementary material for: DiffCoEx: a simple and sensitive method to find differentially coexpressed gene modules
Source: BMC Bioinformatics. 2010 Oct 6;11:497. doi: 10.1186/1471-2105-11-497 (PMC2976757; doi:10.1186/1471-2105-11-497)
Supplement: Additional file 4 — Simulation study showing the sensitivity of DiffCoEx. This file details the result of a simulation study performed to illustrate a scenario in which DiffCoEx will outperform other, less sensitive, methods. [file 1471-2105-11-497-S4.DOC]

Simulation study

**Aim**

The purpose of this simulation is to illustrate the sensitivity of DiffCoEx. We show here that when the dominant correlation structure is stable across conditions, the more subtle changes may be missed by methods that perform clustering based on the correlation structure in one of the two conditions. Moreover, we demonstrate how the dissimilarity metrics used by DiffCoEx can capture module-to-module differential coexpression patterns. The code use to implement this simulation can be found in **Additional File 1**.

**Simulated data**

We present a simulation study involving 1500 genes measured over two different conditions in a total of 200 samples (100 for each condition). The large sample number in the simulations was chosen to reduce the variability between simulation runs and reveal consistent performance differences. Expression data was simulated using random normal distributions and a modular correlation structure was introduced as follows:

1. m1 (red), m2 (black), m3 (blue) and m4 (green) are four coexpressed modules which are stable across both conditions – they should therefore not be detected as *differentially* co-expressed.
2. m5 (yellow) is a module comprised of genes from m1, m2, m3 and m4 which is coexpressed in condition 1 but not in condition 2 (an example of criteria (i) in the main text).
3. m6 (white) is a module which is coexpressed only in condition 1 as another example of criteria (i).
4. m7 (brown) and m8 (pink) are two coexpressed modules which additionally have module-to-module coexpression in condition 2 but not in condition 1 (an example of criteria (ii) from the main text).

The correlation structure was introduced using random vectors drawn from normal distributions that are added to the expression of genes between which we wish to enforce a correlation. The strength of the correlation is controlled by adjusting the variance of the vectors, which are the “seeds” of the correlation modules. An additional sub-modular correlation structure is introduced within the modules using the same approach.

The rest of the genes are colored grey and their expressions follow independent random normal distributions.

**Result with semi-targeted approach:** coXpress (Watson et al. 2005 [1]).

coXpress is an R package which implements a semi-targeted approach: modules are first defined by clustering using correlation as a distance matrix in the first condition, then the modules are tested for changes in coexpression in the second condition.

The results of coXpress on the simulated dataset are summarized below.


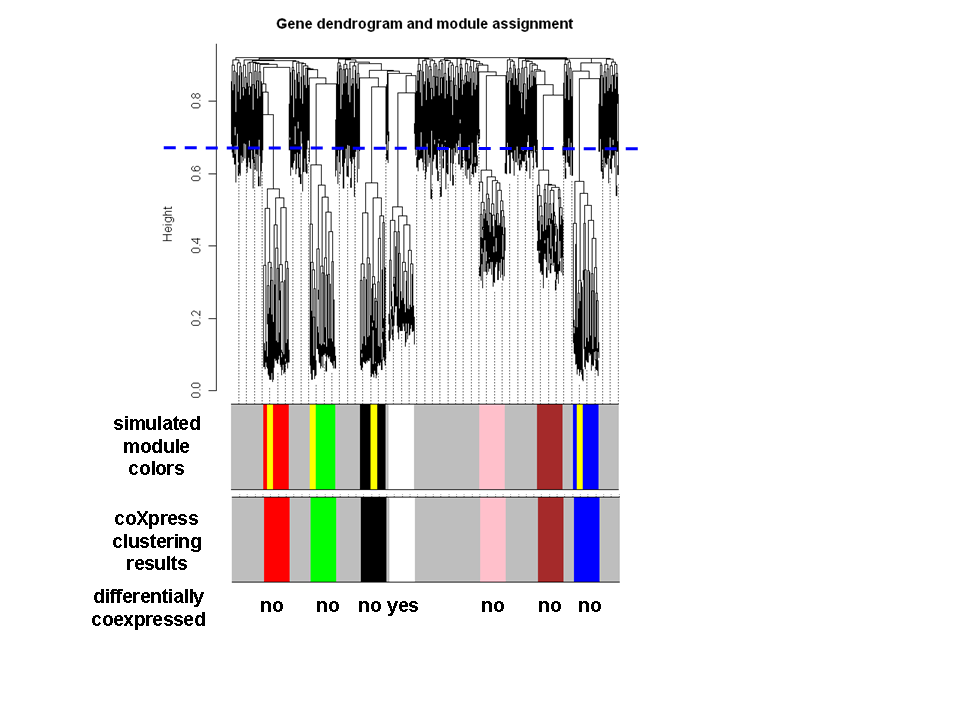


Figure 1 Clustering of the simulated data based on condition 1. The dashed blue line represents the static tree cutting used to define the modules. Below the dendogram, the first color bar represents the modules as they were assigned in the simulation. The bottom color bar represents the modules that are tested by coXpress, of those only the white module is found to be differentially coexpressed.

We see here that coXpress correctly identifies m1, m2, m3 and m4 as modules which are not differentially coexpressed, while the m6 (white) module is correctly identified as differentially coexpressed. However, m5 (yellow) is completely missed by the approach and m7 and m8 are individually tested for differential coexpression (which is not present), but not for module-to-module differential coexpression.

**Results using DiffcoEx**

Applying DiffCoEx on the same simulated dataset yields different results to those shown in **Figure 2**. All three differential coexpression patterns in the dataset are picked up (m5-yellow, m6-white and the module–to-module differential coexpression between the m7-pink and m8-brown modules). Because DiffCoEx works on the difference of correlation matrices, it is able to ignore the dominant but stable correlation structure that is masking the m5-yellow module. Moreover, the use of a topological overlap-based metrics allows the identification of module-to-module differential coexpression between m7-brown and m8-pink: while the correlation between the genes within m7 (or m8) remains stable, the correlation of those genes with the genes from m8 (or m7) drops in condition 2.

The red, blue, black and green modules are (correctly) not reported by DiffcoEx, as these are consistently co-expressed in both conditions and show no *differential* co-expression pattern.


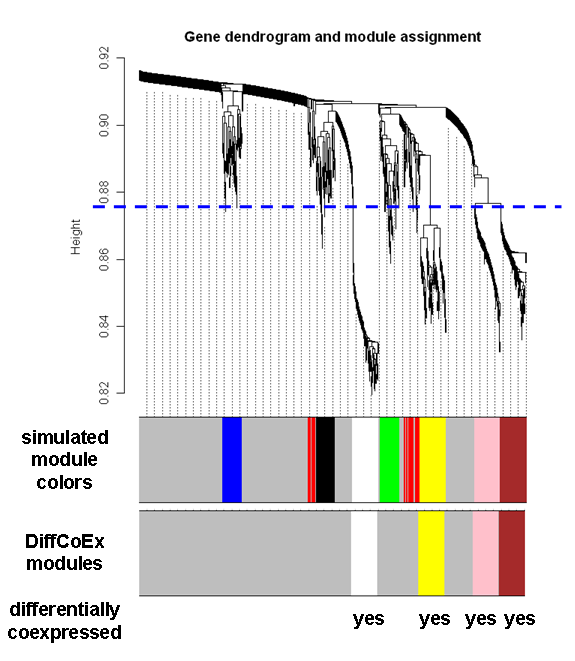


Figure 2 Clustering results using DiffCoEx. The three differential coexpression patterns introduced in the data are correctly identified by DiffCoEx.


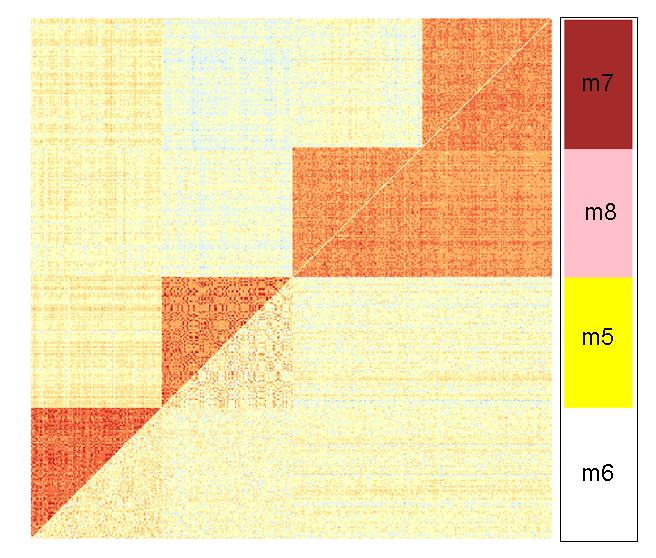


Figure 3 Differential coexpression modules identified by DiffCoEx. The upper diagonal of this heat map represents the correlation between the genes inside the modules identified by DiffCoEx in condition 1, while the lower diagonal shows the correlation of the same genes in condition 2. Both m5 and m6 have clear within-module differential coexpression between the two conditions, while m7 and m8 show module-to-module differential coexpression.

**Conclusion**

This simulated example illustrates some cases for which the sensitivity of DiffCoEx allows more subtle changes to be detected than by existing approaches.

1. Watson M: **CoXpress: differential co-expression in gene expression data**. *BMC Bioinformatics* 2006, **7**:509-509.
